# Retinoic Acid Modulates Immune Differentiation in a Human Small Intestinal In Vitro Model

**DOI:** 10.3390/cells14171300

**Published:** 2025-08-22

**Authors:** Christa Schimpel, Christina Passegger, Carmen Tam-Amersdorfer, Herbert Strobl

**Affiliations:** Division of Immunology, Otto Loewi Research Center for Vascular Biology, Immunology and Inflammation, Medical University of Graz, 8010 Graz, Austria; christina.passegger@medunigraz.at (C.P.); carmen.tam-amersdorfer@medunigraz.at (C.T.-A.); herbert.strobl@medunigraz.at (H.S.)

**Keywords:** small intestine, inflammation, IBD, retinoic acid, intestinal immune response

## Abstract

Retinoic acid (RA) plays a key role in mucosal immune regulation and tolerance, with implications for inflammatory bowel disease (IBD). However, its effects have not been extensively studied in humanized in vitro models that recapitulate epithelial–immune interactions. We established a 3D in vitro small intestinal model composed of three epithelial cell types, naïve CD4^+^ T cells, and monocyte/dendritic cell (M/DC) precursors derived from CD34^+^ umbilical cord blood hematopoietic stem/progenitor cells. The epithelial microenvironment strongly suppressed monocyte/DC differentiation and T cell activation, indicating a regulatory role of epithelial-derived signals. Retinoic acid (RA) priming of M/DC precursors induced CD103^+^CD11b^+^Sirp1α^−^ regulatory DCs and promoted a shift from naive to memory-type T cells. Upon addition of pro-inflammatory cytokines (TNF-α, IFN-γ, IL-1β), the model mimicked an inflamed intestinal state, resulting in CD14^+^CD16^+^ inflammatory monocytes and increased T cell activation (CD25^+^CD69^+^). RA-primed DCs modestly counterbalanced T cell activation and IBD-like responses, even under inflammatory conditions. Flow cytometry and clustering analysis revealed distinct immune cell phenotypes depending on RA exposure and cytokine context. This model provides a reproducible and physiologically relevant human system to study RA-mediated immune programming in the intestinal mucosa and may support the development of novel therapeutic strategies for IBD and related inflammatory conditions. Statistical differences were evaluated using ANOVA with Tukey’s post-hoc test (*n* = 4; *p* < 0.05).

## 1. Introduction

Inflammatory Bowel Disease (IBD), which includes Crohn’s disease and ulcerative colitis, is characterized by chronic inflammation of the gastrointestinal tract [1]. The pathogenesis of IBD is often associated with a disruption in immune homeostasis, leading to an imbalance between pro-inflammatory and regulatory immune responses [2,3]. A key player in maintaining immune balance within the intestinal mucosa is the CD103^+^ dendritic cell (DC) subset [4,5], which is critical for inducing regulatory T cells (Tregs) that mediate immune tolerance and prevent excessive immune activation [6,7].

The small intestine hosts diverse immune cells within organized lymphoid structures, such as Peyer’s patches [8]. Studying immune cell differentiation and function in this dynamic environment is challenging, as current in vitro models often fail to fully recapitulate immune dynamics, especially in chronic inflammation such as IBD [9,10,11]. In particular, dissecting epithelial–immune interactions remains difficult.

Previous in vitro studies, including our own, indicate that intestinal epithelial cells can create a suppressive environment that impairs the differentiation of monocytes into regulatory subsets such as CD103^+^ DCs [12]. These cells are often reduced and functionally impaired in IBD [13,14], contributing to sustained inflammation and immune dysregulation [15,16,17].

To address these limitations, an advanced 3D in vitro model simulating the small intestinal environment was developed. While macrophages have been functionally studied in co-culture with intestinal epithelial cells, studies on ontogeny and local differentiation of M/DCs remain limited [18,19,20]. Crosstalk between epithelial cells, DCs, and T cells, especially under inflammatory conditions, remains insufficiently understood. Published 3D intestinal models, including organoids, typically lack DC sub-lineage differentiation and functional T cell activation.

It remains unclear how circulating blood precursors of intestinal M/DCs are instructed within the epithelial microenvironment, and which signals promote tolerogenic DC differentiation. Although CD14^+^ monocytes can differentiate into CD103^+^ DCs in response to RA, the precise precursor–RA interactions in the intestinal microenvironment remain unclear. Can we establish a 3D human intestinal model mimicking physiological inflammation vs. pathological inflammation characteristic of IBD? Answering this question requires a model system that enables mechanistic interrogation of epithelial–immune communication. We considered addressing this question in a human ex vivo model to be of high medical relevance.

A human 3D model was established, comprising three types of epithelial cells, T cells, and progeny of M/DC precursors. RA-primed M/DCs were successfully integrated and partially counteracted IBD-associated inflammatory cytokines. This study provides new insight into epithelial–immune crosstalk, demonstrating how RA and inflammatory cytokines shape DC and T cell phenotypes in a human-relevant 3D model.

## 2. Materials and Methods

### 2.1. Cell Culture

Caco-2 cells (ACC169, HTB-37 clone; German Collection of Microorganisms and Cell Cultures) were maintained at 37 °C in a humidified incubator with 10% CO_2_, following the protocol described by des Rieux et al. [21]. The culture medium was composed of Dulbecco’s Modified Eagle Medium (DMEM) supplemented with 10% fetal bovine serum (FBS) (Gibco^®^ Thermo Fisher Scientific, Vienna, Austria), 1% non-essential amino acids (NEAA) (Sigma Aldrich, St Louis, MO, USA), and 1% penicillin–streptomycin (PenStrep) (PAA Laboratories, Pasching, Austria). HT29-MTX cells were kindly provided by T. Lesuffleur (INSERM UMR S 938, Paris, France) and were cultivated under the same conditions as Caco-2 cells, i.e., at 37 °C in a water-saturated atmosphere with 10% CO_2_, using identical complete medium [22]. Raji B cells (provided by R. Fuchs, Medical University of Graz, Austria) were propagated in RPMI 1640 medium enriched with 10% FBS, 1% NEAA, 1% L-glutamine, and 1% penicillin–streptomycin, maintained at 37 °C with 5% CO_2_, according to established protocols [23]. Caco-2 and HT29-MTX cell lines were passaged weekly using trypsin–EDTA (0.25%, 0.53 mM) and reseeded at a density of 4 × 10^5^ cells per 75 cm^2^ culture flask. Raji B cells were also subcultured once a week by diluting the cell suspension with fresh medium to maintain a concentration of approximately 1 × 10^6^ cells per 75 cm^2^ flask. Medium replacement was performed every second day.

### 2.2. Cord Blood Collection and Cell Isolation

Cord blood (CB) samples were collected during healthy full-term deliveries with written informed consent and approval by the Ethics Committee of the Medical University of Graz (approval date: July 2014). Mononuclear cells were isolated from cord blood samples within 10 h of collection using a discontinuous density gradient centrifugation with Ficoll/Hypaque. Subsequently, CD34^+^ hematopoietic stem and progenitor cells were purified from the mononuclear cell fraction using the EasySep™ Human Cord Blood CD34 Positive Selection Kit II (STEMCELL Technologies, Cologne, Germany), following the manufacturer’s instructions. The purity of isolated CD34^+^ cells was routinely >90%, as confirmed by flow cytometry. After isolation, cells were expanded for 3 days in X-VIVO™ 15 Medium (Lonza) supplemented with 1% GlutaMAX, 1% PenStrep, and 50 ng/mL stem cell factor (SCF), Fms-related tyrosine kinase 3 ligand (FLT3-L), and thrombopoietin (TPO) before they were used for the setup of the small intestinal model.

### 2.3. CD4^+^ T Cell Isolation

CD4^+^ T cells were enriched from peripheral blood mononuclear cells using the MagniSort™ Human CD4 T Cell Enrichment Kit (Thermo Fisher Scientific, Vienna, Austria), following the manufacturer’s protocol. This kit employs a negative selection strategy, in which a cocktail of antibodies targeting non-CD4^+^ T cell populations (including CD8, CD11b, CD14, CD16, CD19, CD20, CD36, CD56, CD123, CD235a, and γδ TCR) is used to deplete unwanted cells, thereby yielding an untouched and highly pure CD4^+^ T cell population. The purity was routinely >95%, as confirmed by CD3/CD4 staining and FACS analysis.

### 2.4. Preliminary Experiments with Retinoic Acid (RA)

Before using RA-treated precursors for the in vitro setup, preliminary experiments were conducted to determine an appropriate RA concentration that promotes CD103 differentiation while avoiding toxicity. For this, CD34^+^ precursor cells were cultured in 24-well plates for 5 days in expansion mix (as described above), with varying concentrations of RA (2 µM, 10 µM, 20 µM, 40 µM). Control groups consisted of M/DC precursors cultured in the same medium without RA. Cell differentiation was analyzed by immunophenotyping (i.e., CD marker expression). To exclude solvent-induced cytotoxicity during RA pre-treatment, DMSO-only controls were included (final DMSO concentration: 0.03%). Cytotoxicity was evaluated using the LDH Cytotoxicity Assay (Thermo Fisher Scientific), following the manufacturer’s protocol. LDH release in DMSO-only controls (6.3 ± 1.1%) was comparable to untreated controls (4.8 ± 0.9%), and significantly lower than the positive control (1% Triton X-100, 100%). These results confirm that the solvent concentration used for RA delivery was non-toxic to M/DC precursors under the applied conditions.

### 2.5. PKH Staining

CD34^+^cells were fluorescently labeled using the PKH26GL staining kit from Sigma Aldrich, as described in Schimpel et al. [12]. Cells (5 × 10^6^ to 1 × 10^7^ cells/mL) were centrifuged (5 min, 1280 rpm), and the pellet was resuspended in 250 µL Diluent C. The cell suspension was briefly vortexed (for a maximum of 5 s at 1400 rpm) while adding 250 µL of freshly prepared dye solution and incubated for 30 s at room temperature. Staining was quenched with 2 mL heat-inactivated FBS, followed by a 1 min incubation and centrifugation. Cells were then washed twice in complete DMEM, centrifuged, and finally resuspended in fresh DMEM.

### 2.6. Setup of the Intestinal Cell Culture Models

To set up the triple culture system consisting of Caco-2, HT29-MTX, and immune cells, a total of 1 × 10^5^ intestinal epithelial cells per well were seeded at a 7:3 ratio of Caco-2 to HT29-MTX, together with 1 × 10^5^ immune cells (PKH-labeled CD34^+^ cells, RA-pretreated CD34^+^ cells, and bulk CD4^+^ T cells). Cells were suspended in 1.5% GrowDex^®^ hydrogel (UPM Biomedicals, Helsinki, Finland) to yield a final concentration of 0.75%, and 100 µL of this mixture was applied onto polycarbonate Transwell^®^ filters (3 µm pore size, 0.33 cm^2^ area; Corning Inc., Corning, NY, USA). Cultures were incubated under standard conditions for 14–16 days to allow epithelial and immune cell integration. To stimulate M cell differentiation, 3 × 10^5^ Raji B cells, suspended in complete DMEM, were added to the basolateral chamber and maintained for an additional 4–5 days, with media changes performed every second day. To mimic an inflamed state resembling IBD, cells were treated with IL-1β (50 ng/mL), TNF-α, and IFN-γ (100 ng/mL) on days 14, 16, and 19. All experiments were performed with four independent biological replicates (*n* = 4), each derived from a separate donor. Depending on the specific assay, technical triplicates were included for each replicate.

### 2.7. Cell Harvesting and Flow Cytometry

Cells were harvested using GrowDase™ (UPM Biomedicals), a purified mixture of cellulase enzymes designed to break down the nanocellulose fibrils in GrowDex^®^ into soluble glucose. The enzyme was mixed with the sample and incubated at 37 °C overnight, allowing the GrowDex^®^ to be fully converted into glucose while preserving cell structure, viability, and functionality.

Following harvest, human cells were washed in phosphate-buffered saline (PBS) and subsequently resuspended in staining buffer. To prevent nonspecific binding, Fc receptors were blocked by incubating the cells with human serum for 15 min on ice. Surface staining was then performed on ice for a minimum of 30 min using fluorochrome-conjugated monoclonal antibodies. The full list of antibodies used for flow cytometric analysis is provided in Table S1 (Supplementary Information).

### 2.8. Data Acquisition and Analysis

Data were acquired on an LSR Fortessa instrument (BD Biosciences, San Jose, CA, USA) and analyzed using FlowJo software (Version 10.7). Forward versus side scatter (FSC vs. SSC) gating was used to identify cells of interest based on size and granularity, whereas side scatter height versus side scatter width gating was used for doublet exclusion. Starting from live cell population and single cells, gates were applied where only PKH-stained cells were used for the setup of further gates.

Clustering was performed on PKH-stained cells (RA-treated vs. untreated), including XShift, t-SNE dimensionality reduction, and the Cluster Explorer (as previously described [12]).

### 2.9. Statistical Analysis

Statistical analyses were performed using GraphPad Prism 10. All data are presented as mean ± standard deviation (SD). Differences between groups were evaluated using one-way ANOVA, followed by Tukey’s multiple comparison test. A *p*-value of <0.05 was considered statistically significant. Each condition was tested in four independent biological replicates (*n* = 4), unless indicated otherwise.

### 2.10. Ethics

Umbilical cord blood was obtained from healthy donors during full-term delivery at the University Clinic for Gynaecology and Obstetrics (Frauenklinik), Hospital Graz, Austria (ethical approval: 26-520 ex 13/14, approval date: July 2014).

Buffy coats were purchased from the Department of Transfusion Medicine, Hospital Graz, Austria.

## 3. Results

### 3.1. Creation of a 3D Intestinal Model Permissive for the Generation of Inflammatory Monocytes and Activated Memory T Cells

**Figure 1:**
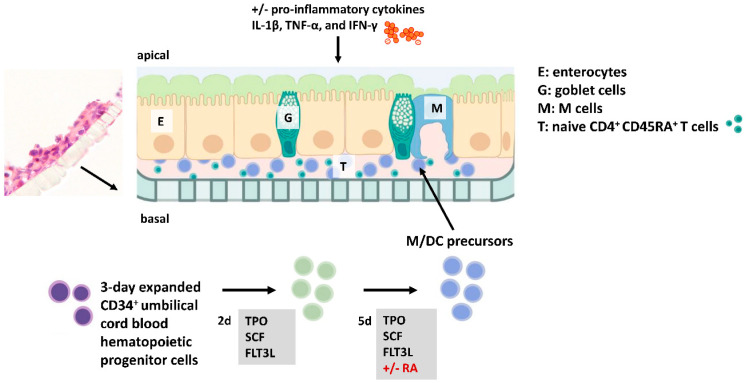
Experimental setup of the intestinal in vitro model. Immune cells (i.e., PKH-stained M/DC precursors and CD4^+^ T cells) and intestinal epithelial cells (IECs) (at a ratio of 7:3 Caco-2:HT29-MTX) are mixed and seeded to the upper side of filters within an animal-free birch-based cellulose hydrogel (GrowDex^®^). This setup allows systematic testing of additional parameters such as IBD-associated pro-inflammatory cytokines and RA-priming of M/DC precursors. Flow cytometry (FACS) was applied to examine the differentiation markers and activation profiles of immune cells across both cytokine-deficient and cytokine-complemented conditions.

We previously established a 3D in vitro model of the small intestine to study the microenvironmental signals required for DC differentiation from CD34^+^ hematopoietic progenitor cells (HPCs). In this model, 3-day pre-expanded (frozen) CD34^+^ umbilical cord blood cells were expanded for 7 days in the presence of FLT3L, SCF, and TPO to generate M/DC precursor cells. These M/DC precursors lacked substantial expression of terminal M/DC markers, but had the potential to rapidly differentiate into CD14^+^CD11b^+^ monocytes and CD1a^+^ dendritic cells upon sub-culturing in GM/TNF-α/FLT3L-supplemented suspension cultures [24]. The fate of these functional M/DC precursors was studied in response to additional signals in the 3D model (Figure 1). First, we introduced naïve allogeneic CD4^+^ T cells to simulate crosstalk between T cells and M/DCs within the intestinal mucosa.

### 3.2. Epithelial Cells and T Cells Fail to Promote M/DC Precursor Cell Differentiation

**Figure 2:**
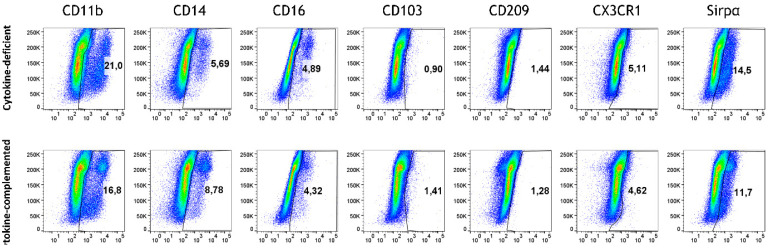
Monocyte differentiation in the cytokine-deficient vs. cytokine-complemented model. Flow cytometry dot plots represent PKH^+^ cells (side scatter; SSC) vs. expression levels in % of indicated differentiation markers; data are pooled from 4 experiments; for each experiment, a different CD34^+^ and CD4^+^ T cell donor was used (biological replicates: *n* = 4).

Exposing dye-labeled M/DC precursor cells to epithelial cells in the 3D intestinal model for extended periods of time did not result in the induction of M/DC marker characteristics (Figure 2, upper panel, “cytokine-deficient”). Only minor percentages of cells acquired CD11b, CD14, CD16, and Sirpα, and other macrophage/DC markers (CD103, CD209, CXRCR1) were not detected. This reductionist approach permitted us to introduce additional intestinal factors to the 3D epithelial/T cell model, such as pro-inflammatory cytokines and RA priming of M/DC precursors.

### 3.3. Pro-Inflammatory Cytokines Fail to Rescue CD103^+^ DC Differentiation but Promote Inflammatory Monocyte Differentiation in the 3D Model

**Figure 3:**
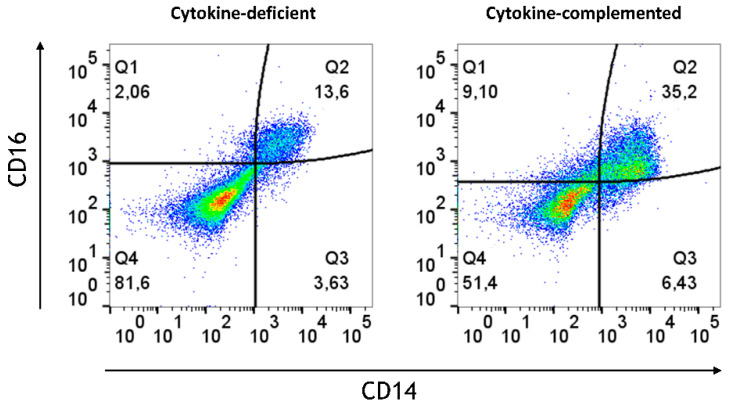
Increased intermediate monocytes in the cytokine-complemented model. Dot plots representing CD11b^+^ cells in the cytokine-complemented vs. cytokine-deficient (control) model. Plots depict CD16 expression levels in % vs. CD14 expression levels in %; data are pooled from 4 experiments; for each experiment, a different CD34^+^ and CD4^+^ T cell donor was used (biological replicates: *n* = 4). These three markers are typically expressed by monocytes and also serve to further subclassify the monocyte subsets. Q1 = CD11b^+^CD16^+^ (non-classical/patrolling monocytes; Q2 = CD11b^+^CD14^+^CD16^+^ (intermediate monocytes); Q3 = CD11b^+^CD14^+^ (classical monocytes); Q4 = CD11b^+^.

In order to mimic steady-state intestinal physiological inflammation and IBD, pro-inflammatory signals were added; specifically, we addressed whether these might bypass immunosuppression in the epithelial/T cell 3D model. Thus, IL-1β, TNF-α, and IFN-γ [25] (termed “cytokine-complemented”) were added to the 3D model, and the consequences on M/DC differentiation and T cell activation were analyzed. Despite these additional factors, CD103^+^ DCs were absent in both cytokine-deficient and cytokine-complemented models, and only minimal expression of monocyte/DC markers (CD11b, CD14, CD16, Sirpα) was observed (Figure 2). Nevertheless, a notable increase in intermediate monocytes (CD11b^+^CD14^+^CD16^+^) was observed in the cytokine-complemented model (Figure 3). These monocytes, known for their pro-inflammatory potential, were significantly elevated compared to the cytokine-deficient condition; specifically, cells in Q2 increased from 13.3 ± 0.29% to 35.2 ± 0.22% (*p* < 0.0001, *n* = 4), and such cells can also be observed in IBD lesions [26,27]. This indicates that cytokines alone drive inflammatory skewing but are insufficient to instruct tolerogenic DC programming in the absence of specific tissue-derived signals.

### 3.4. Increased Expression of Antigen-Presenting Cell (APC) Activation Markers and T Cell Activation in Response to Pro-Inflammatory Cytokines

**Figure 4:**
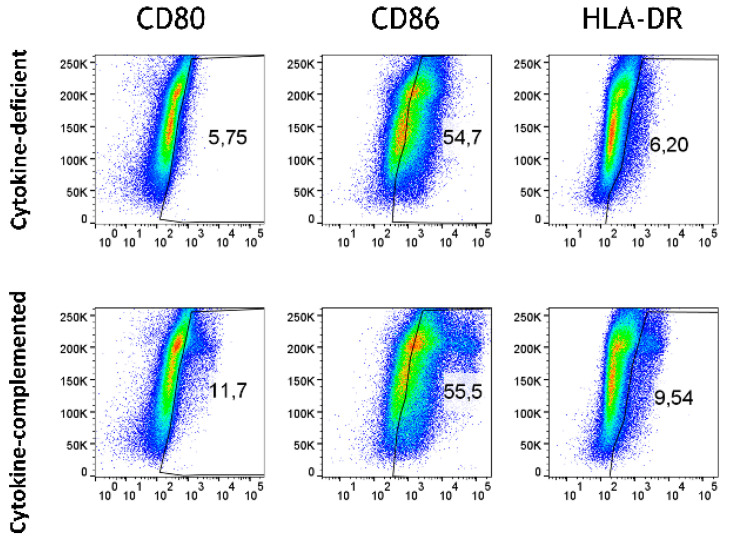
Antigen-presenting cell activation in the cytokine-deficient vs. cytokine-complemented model. Plots depict side scatter (SSC) vs. expression levels in % of indicated activation markers. Data are pooled from 4 experiments; for each experiment, a different CD34^+^ and CD4^+^ T cell donor was used (biological replicates: *n* = 4).

**Figure 5:**
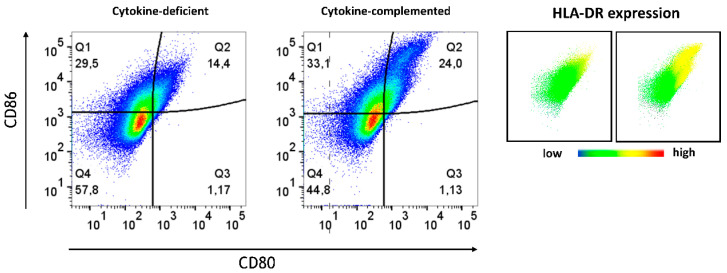
Plots depict CD86 expression levels in % vs. CD80 expression levels in %; data are pooled from 4 experiments; for each experiment, a different CD34^+^ and CD4^+^ T cell donor was used (biological replicates: *n* = 4). HLA-DR expression is shown on the right. Coloring corresponds to expression intensity, with red indicating high expression and blue indicating absence of the marker.

Beyond differentiation, the expression of APC activation markers was analyzed in both models. Whereas in the cytokine-deficient condition, low levels of APC activation markers, including CD80, CD86, and HLA-DR, were detected, the cytokine-complemented model led to an increase in CD80^+^ CD86^+^ HLA-DR^+^ cells, indicative of heightened antigen presentation capacity (Figure 4 and Figure 5).

**Figure 6:**
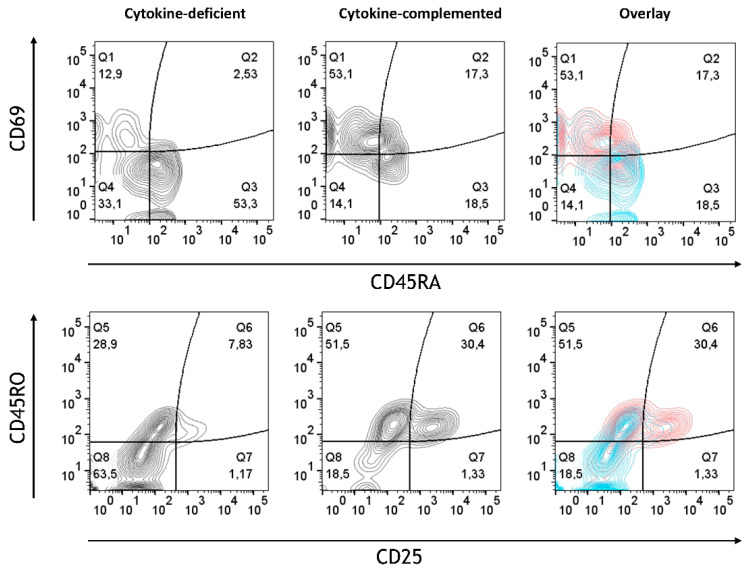
T cell activation in cytokine-deficient vs. cytokine-complemented model. Dot plots representing CD4^+^ T cells in the healthy vs. control model. Plots depict CD69 expression levels in % vs. CD45RA expression levels in % as well as CD45RO expression levels in % vs. CD25 expression levels in %; data are pooled from 4 experiments; for each experiment, a different CD34^+^ and CD4^+^ T cell donor was used (biological replicates: *n* = 4).

Whereas in the cytokine-deficient model, most T cells retained their non-activated naïve phenotypic characteristics (CD45RA^+^CD45RO^−^CD25^−^CD69^−^), the cytokine-complemented model resulted in increased expression of CD25 and CD69, alongside a shift from naïve (CD45RA^+^) to memory-like (CD45RO^+^) T cells (Figure 6). Quantification of combined T cell phenotypes confirmed a highly significant shift: the frequency of CD45RA^+^CD45RO^−^CD25^−^CD69^−^ cells dropped from 53.70 ± 4.09% to 18.95 ± 2.87%, while CD45RA^−^CD45RO^+^CD25^+^CD69^+^ cells increased from 7.30 ± 1.52% to 30.58 ± 3.54% (both *p* < 0.0001, *n* = 4). This reflects sustained immune cell activation, similar to what is seen in chronic inflammatory conditions such as IBD [28]. These findings indicate that cytokine exposure promotes functional T cell activation in the 3D environment, highlighting signaling-driven crosstalk between tissue-conditioned myeloid cells and naïve T cells.

### 3.5. Retinoic Acid (RA) Promotes the Generation of CD103^+^ DCs at the Expense of Sirpα^+^ DCs from M/DC Precursor Cells in Suspension Cultures

**Figure 7:**
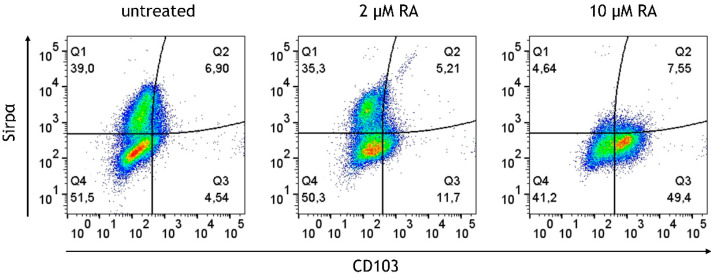
Dose-dependent induction and upregulation of CD103 on M/DC precursors in response to RA. Dot plots represent gated CD11b^+^ cells generated in the absence or presence of 2 µM or 10 µM RA. Plots depict Sirpα expression levels in % vs. CD103 expression levels in %; data are pooled from 3 experiments; for each experiment, a different CD34^+^ donor was used (biological replicates: *n* = 3).

The human small intestine harbors CD103^+^ DCs. In vitro studies previously demonstrated that CD103 can be induced in monocytes in response to RA stimulation [29]. The M/DC precursor generation cultures, derived from CD34^+^ hematopoietic progenitor cells (HPCs), encompass cells at different stages of monocyte and conventional DC differentiation, and to the best of our knowledge, the effects of RA in such HPC cultures have not been studied. Since intestinal M/DC lineage cells are constantly replenished from candidate circulating blood M/cDC precursors, and undergo differentiation in response to the intestinal microenvironment, these cells were exposed to RA prior to integration in the epithelial/T cell 3D model and followed their cell differentiation. The above-described M/DC precursor cell generation cultures were supplemented with different concentrations of RA (2 μM, 10 μM, 20 μM, and 40 μM) for 5 days. The generated cells were monitored for CD103 in combination with CD11b and Sirpα^+^ to identify CD103^+^CD11b^+^Sirpα^−^ and CD103^+^CD11b^+^Sirpα^+^ DCs, known to be present in the human small intestine [30]. This approach sought to mimic in vivo exposure of immigrating M/DC precursors to local RA known to occur at high concentrations in the small intestine, arising from the metabolism of dietary vitamin A [31]. In the absence of RA, a substantial portion of cells acquired the monocyte/cDC2-associated marker Sirp1α. RA treatment of M/DC precursors resulted in the dose-dependent upregulation of CD103 at the expense of Sirp1α among gated CD11b^+^ cells, suggesting that RA alters DC sublineage differentiation of M/DC precursors (Figure 7). Quantification of the CD103^+^CD11b^+^Sirpα^+^ subset (Q3) showed a significant increase from 4.05 ± 0.90% at 0 µM RA to 10.92 ± 2.68% at 2 µM RA and 47.10 ± 4.75% at 10 µM RA (*p* < 0.0001, one-way ANOVA, *n* = 4). Highly granulated Sirpα^+^ cells dropped with increased RA concentrations, indicative of repressed monocyte/cDC2 differentiation. This shift suggests that CD103^+^ cells originate from monocyte/cDC2 committed cells in response to RA. These findings support the role of RA as a soluble instructive signal that modulates DC lineage fate decisions during mucosal immune programming.

### 3.6. RA-Generated CD103^+^ DCs and CX3CR1^+^ Macrophages Remain Stable After Integration into the Cytokine-Deficient 3D Intestinal Model

**Figure 8:**
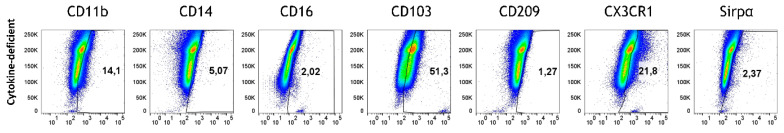
The fate of RA-pre-treated M/DC precursors in the cytokine-deficient model. Flow cytometry dot plots represent PKH^+^ cells (side scatter; SSC) vs. expression levels in % of indicated differentiation markers; data are pooled from 4 experiments; for each experiment, a different CD34^+^ and CD4^+^ T cell donor was used (biological replicates: *n* = 4).

Flow cytometric analysis revealed that RA-primed M/DC precursors retained a stable CD103^+^Sirpα^−^ DC phenotype upon integration into the model (Figure 8). This suggests that RA-induced programming is sufficient to imprint a DC lineage identity that remains stable within the epithelial/T cell environment, even in the absence of continued RA exposure.

**Figure 9:**
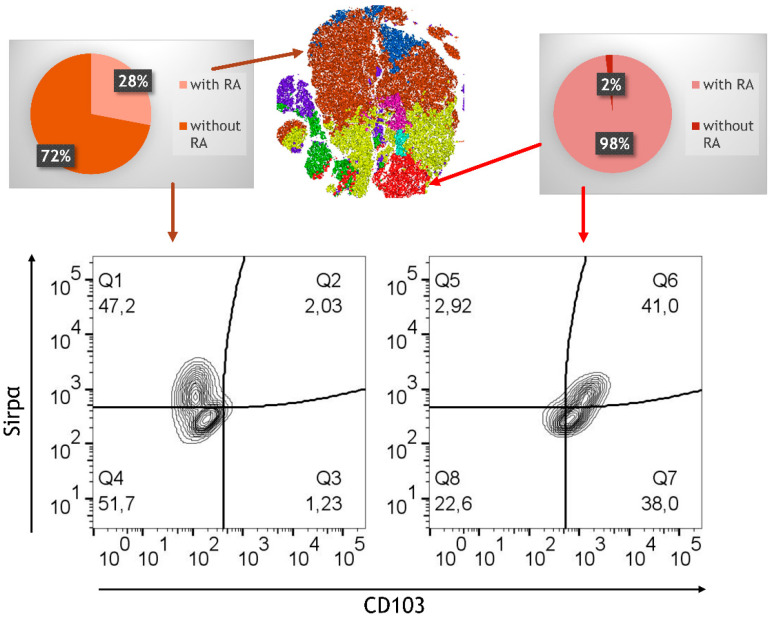
Phenotypic analysis via FlowJo. tSNE analysis followed by clustering of flow cytometry data for the RA-treated model and the untreated model; data (10^5^ PKH^+^ cells from RA-treated model and untreated control) are pooled from 4 experiments; for each experiment, a different CD34^+^ and CD4^+^ T cell donor was used (biological replicates: *n* = 4). Pie charts showing the distribution (in %) of the RA-treated versus untreated model. Expression of Sirpα and CD103 is shown in two selected FACS representing CD11b^+^.

In addition to CD103^+^ DCs, CX3CR1^+^ cells—indicative of monocyte-derived macrophages—were detected, albeit in lower frequencies compared to CD103^+^ DCs. To gain deeper insights into the cellular changes induced by RA, a graph-based clustering analysis using XShift was used, followed by t-stochastic neighbour embedding (tSNE) for dimensionality reduction. This analysis revealed the presence of eight distinct cell clusters within the RA-treated and untreated control model (Figure 9). This clustering allowed us to discern the phenotypic diversity of M/DC precursors pre-treated (or not) with RA after integration into the 3D model. In the untreated model, approximately 75% of PKH-stained cells were located in a large brown cluster, which exhibited high expression of monocyte characteristics, including CD11b, CD14, and Sirpα. These cells, however, lacked substantial expression of markers associated with DCs and macrophages, such as CD103 and CX3CR1, confirming their limited differentiation potential. Other smaller clusters (blue, purple) in the untreated model exhibited lower levels of CD14 and CD16; however, overall, the cell populations were predominantly monocyte-like, lacking functional specialization.

In contrast, the RA-treated model exhibited a marked shift in phenotypic distribution. Monocyte-characteristic clusters were notably reduced, replaced by a more heterogeneous population expressing CD103 and CX3CR1, markers associated with regulatory DCs and macrophages critical to gut immune tolerance. The appearance of this heterogeneity underscores RA’s effectiveness in guiding immune differentiation toward phenotypes conducive to immune balance. Strikingly, the RA-treated model revealed the presence of a unique population of cells characterized by high expression of CD103, CD11b, and Sirpα. These cells, which we refer to as tolerogenic DCs, were exclusively found in the RA-treated model and comprised approximately 15% of the precursor cells in a distinct cluster (red cluster). This highly specialized population of CD103^+^CD11b^+^Sirpα^+^ DCs is known for its ability to promote the differentiation of regulatory T cells (Tregs), a critical mechanism for maintaining immune tolerance in the gut [32]. In addition to the development of tolerogenic DCs, RA also promoted the differentiation of CX3CR1^high^CD103^dim^CD11b^high^ macrophages, which were located in a separate cluster (yellow cluster). CX3CR1^+^CD11b^high^ cells, phenotypically similar to regulatory macrophages, were distributed across three smaller clusters (green, turquoise, pink) and were further distinguished by variable expression levels of CD14 and CD16. The diversity of these immune cells suggests that RA not only supports the development of tolerogenic DCs but also fosters the generation of macrophages with potential anti-inflammatory and homeostatic functions.

### 3.7. Limited Induction of Regulatory Cells and Persistent Inflammatory Monocytes in the RA-Primed Cytokine-Complemented Model

**Figure 10:**
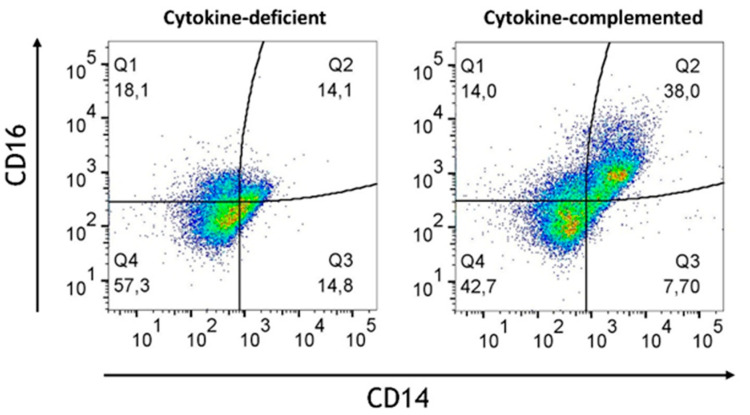
Increased pro-inflammatory CD14^+^CD16^+^monocytes in the cytokine-complemented (inflamed) model. Dot plots representing CD11b^+^ cells in the cytokine-deficient and cytokine-complemented (inflamed) model, both treated with RA. Plots depict CD16 expression levels in % vs. CD14 expression levels in %; data are pooled from 4 experiments; for each experiment, a different CD34^+^ and CD4^+^ T cell donor was used (biological replicates: *n* = 4).

Given the challenges observed in the differentiation of immune cells without RA, especially the lack of regulatory cells, it was investigated how RA-primed M/DC precursors might influence the immune environment under both cytokine-complemented and cytokine-deficient conditions. Our hypothesis was that RA priming might potentially help re-establish immune homeostasis by adjusting the balance between pro-inflammatory and regulatory immune cells. To test this, M/DC precursors were pre-treated with RA, then stained with PKH dye following co-culture with allogeneic naïve CD4^+^ T cells in the intestinal model. Flow cytometric analysis at day 21 post-initiation provided detailed insights into RA’s modulatory effects across both inflammatory and homeostatic contexts. Across both models, RA priming resulted in notable changes in immune cell populations. Despite RA pre-treatment, inflammatory cytokines led to a persistent population of pro-inflammatory monocytes (CD14^+^CD16^+^), which are commonly associated with inflammatory responses in diseases such as IBD (Figure 10). The levels of these pro-inflammatory monocytes were notably higher in the cytokine-complemented model compared to the cytokine-deficient RA-pre-treated model, indicating that the inflamed environment continues to favour the expansion of inflammatory immune cells despite RA’s immunomodulatory effects. Quantification confirmed a significant increase in pro-inflammatory CD14^+^CD16^+^ monocytes in the cytokine-complemented model (35.2 ± 6.6%) compared to the cytokine-deficient RA-pre-treated model (13.3 ± 2.1%; *p* < 0.01, *n* = 4).

This persistence of inflammatory monocytes, despite RA conditioning, suggests that local inflammatory cytokine signaling overrides tolerogenic differentiation signals at the myeloid level.

**Figure 11:**
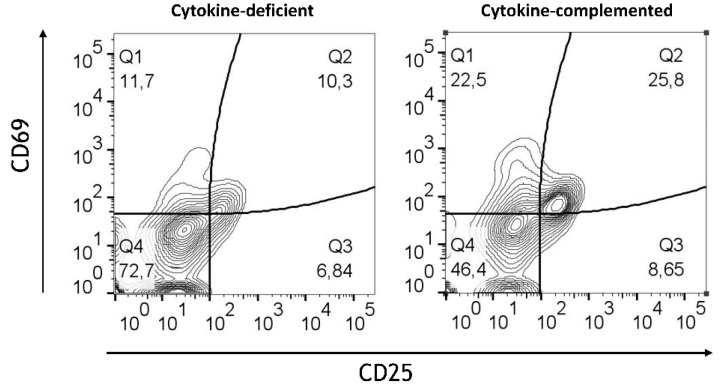
T cell activation in cytokine-deficient vs. cytokine-complemented models. Dot plots representing CD4^+^T CD45RO^+^ cells in the healthy vs. control models. Plots depict CD69 expression levels in % vs. CD25 expression levels in %; data are pooled from 4 experiments; for each experiment, a different CD34^+^ and CD4^+^ T cell donor was used (biological replicates: *n* = 4).

On the other hand, a slight reduction in the expression of regulatory markers such as CD103 and CX3CR1 was detected in the inflamed model, suggesting that RA’s regulatory effects may be attenuated in an inflammatory context. Consistent with this notion, under both conditions, high expression of CD45RO by T cells was observed. Nevertheless, quantification confirmed a significant increase in activated CD45RO^+^ T cells co-expressing CD25 and CD69 in the cytokine-complemented model (28.3 ± 6.1%) compared to the cytokine-deficient RA-pre-treated model (12.5 ± 3.9%; *p* < 0.01, *n* = 4), indicating ongoing T cell activation despite the presence of RA-induced M/DCs (Figure 11).

This analysis highlights that while RA can promote regulatory immune cell differentiation and memory T cell maintenance, its immunomodulatory effects are limited in the cytokine-complemented inflamed model, which continues to support inflammatory cell activation and T cell engagement.

## 4. Discussion

It was demonstrated that intestinal epithelial cells in the absence of inflammatory cytokines fail to support monocyte/macrophage and DC differentiation from M/DC precursors. Whereas the addition of pro-inflammatory cytokines IL-1β, TNF-α, and IFN-γ led to an increase in pro-inflammatory monocytes (CD11b^+^CD14^+^CD16^+^), CD103^+^ DC generation strictly relied on RA-pre-treatment of M/DC precursors. RA dose dependently promoted CD103 at the expense of Sirpα by CD11b^+^ DCs generated from M/DC precursors, consistent with previously described tolerogenic DCs. These cells retained their phenotypic characteristics upon integration into the 3D epithelial model. RA priming of M/DC precursors also promoted CX3CR1^+^ macrophage differentiation. Whereas the integration of RA-pre-treated M/DC precursors into the 3D model counteracted inflammation, as evidenced by a reduced % of activated T cells, inflammatory cytokines led to a reduced % of regulatory CD103^+^ DCs. These observations highlight the robustness of the model in recapitulating homeostatic vs. inflammatory immune responses in a human-specific intestinal context.

Our demonstration of elevated numbers of inflammatory monocytes in response to IL-1β, TNF-α, and IFN-γ is consistent with the previously observed expansion of intermediate monocytes during a heightened pro-inflammatory intestinal response [27]. These pro-inflammatory monocytes are associated with enhanced antigen presentation, as evidenced by the upregulation of APC markers (CD80, CD86, and HLA-DR) [33,34,35], as also seen in this study. T cell activation (CD25, CD69) was enhanced in the cytokine-complemented model, reflecting sustained immune activation. This T cell response appears to be tightly linked to the inflammatory programming of myeloid cells, suggesting a bidirectional signaling axis between cytokine-instructed myeloid subsets and T cell effectors. The shift from naïve (CD45RA^+^) to memory T cells (CD45RO^+^) further indicates that chronic inflammation drives T cell activation and differentiation, contributing to the perpetuation of the inflammatory cycle. These results are consistent with findings by Tindemans et al. [28], who reported increased CD45RO^+^ memory T cells and CD69 expression in chronic inflammation. Vice versa, the observed reduction in CD45RA^+^ immature/naïve T cells might indicate a potential loss of immune tolerance [36]. The cytokine-mediated elevated expression of CD69 in T cells integrated in the model is consistent with a functional role of CD69 in intestinal inflammation [37,38,39]. Similarly, CD25, a marker crucial for T cell activation and IL-2 binding, was upregulated in the inflamed model. This finding aligns with its role in IBD, where CD25 is essential for T cell function and inflammation modulation. CD25 is primarily expressed on activated effector and regulatory T cells [40]. T cells lacking CD25 exhibited reduced suppressive activity, leading to an increase in inflammation within the intestinal tissue. Furthermore, blocking CD25-mediated IL-2 signaling can effectively reduce intestinal inflammation in preclinical models of IBD [41]. Therefore, the observed upregulation of CD25 in the cytokine-complemented in vitro model is in line with the established understanding of CD25’s involvement in T cell activation, survival, and the modulation of inflammatory responses in the context of intestinal inflammation seen in conditions such as IBD.

A key finding of this study was that RA addition dose-dependently upregulated CD103 expression by CD11b^+^ cells generated from CD34^+^ cell-derived M/DC precursors in suspension cultures. Notably, only the CD11b^+^CD103^+^ DCs in the intestinal lamina propria are capable of priming Treg cells and maintaining Th17 cells, which are crucial for intestinal immune homeostasis [42]. As RA concentrations increased, a corresponding decrease in the population of high-granulated Sirpα^+^ cells was found, consistent with the notion that Sirpα may act as a negative regulator in DC activation and homeostasis in CD11b^+^CD103^+^ cells. Sirpα deficiency enhanced the generation of CD11b^+^CD103^+^ DCs [43], and Sirpα-deficient mice exhibited an increased capacity for generating CD11b^+^CD103^+^ DCs, thus underlining Sirpα’s inhibitory role in DC regulation and differentiation. The modulation of these DC subsets by RA may offer a pathway to enhance immune tolerance while reducing inflammatory immune subsets that contribute to chronic intestinal inflammation.

RA-pre-treated and non-treated M/DC precursors were integrated into the 3D intestinal model. It was shown that RA-induced CD103^+^ dendritic cells and CX3CR1^+^ macrophages remain stable within the cytokine-deficient 3D intestinal model. Therefore, once induced by RA, regulatory DCs may remain stable within the epithelial microenvironment without further RA stimulation. This finding highlights the persistence of RA-driven differentiation cues, even in the absence of continued exposure, and underscores the instructive character of early signaling events. The variability in macrophage differentiation in the t-SNE clustering analysis suggests a complex interaction between RA and monocyte-derived cells, indicating a need for further investigation into how RA modulates these pathways under both homeostatic and inflammatory conditions.

Unexpectedly, RA-primed M/DCs induced CD45RO^+^ memory-like T cells without robust CD25 expression. Thus, CD103^+^ DCs might promote activated CD45RO memory-like T cells. Although CD103^+^ DCs are known to promote Treg differentiation in vivo, this model did not include additional readouts such as FOXP3 or CD127 to identify functional Tregs; thus, we interpret the CD45RO^+^CD25^+^ T cells observed in the model as memory-like, while acknowledging that regulatory subsets may also be represented. At first sight, these findings might therefore conflict with previous data demonstrating that RA promotes regulatory T cell (Treg) differentiation through the induction of CD103^+^ DCs [44,45,46,47,48,49]. It has to be considered that RA was not directly added to the 3D model, since prolonged RA exposure might alter 3D epithelial layers. Nevertheless, this model might be suitable for studying the effects of time-restricted RA stimulation on DC-dependent Treg generation.

In the cytokine-complemented model, percentages of CD103^+^ DCs and CX3CR1^+^ macrophages were notably lower than in the cytokine-deficient model. Therefore, pro-inflammatory cytokines reduce the frequencies of these cells. Reduced CX3CR1 expression in this model suggests impaired chemokine signaling, which could affect the recruitment of CX3CR1-expressing cells such as monocytes, macrophages, and DCs. As a chemokine receptor, CX3CR1 is essential for cell migration and adhesion, so its reduced expression may hamper the immune system’s effectiveness in managing chronic inflammation [50,51]. This highlights how inflammatory signaling networks can override homeostatic cues and reshape the immunological landscape toward a chronic activation profile.

Conversely, percentages of CD14^+^CD16^+^ monocytes remained unaltered. These cells are associated with chronic inflammatory conditions, including IBD. The addition of pro-inflammatory cytokines led to the generation of CD45RO^+^ activated T cells (CD25^+^ and CD69^+^). RA-pre-treated M/DC precursors only slightly reduced T cell activation. This mirrors the difficulty of counteracting persistent inflammatory pathways in IBD [52,53]. In conclusion, RA-primed regulatory immune cells may possess only limited potential in reversing the pro-inflammatory state in IBD.

Compared to other human models, our 3D system holds several advantages. Organoids, while suitable for epithelial biology, lack immune components and DC/T cell functionality [54,55]. Mouse models, although widely used, do not recapitulate human-specific immune regulation. Moreover, our system eliminates age-related variables common in patient-derived materials, as immune differentiation can vary with age [56,57]. The Transwell-based setup mimics distinct apical/basolateral compartments, enabling studies of nutrient transport and epithelial–immune cell interaction. Although barrier integrity was not the primary focus of this study, transepithelial electrical resistance (TEER) was assessed in a preliminary experiment to verify monolayer confluence and epithelial integrity. TEER values of 300–450 Ω·cm^2^ were recorded under cytokine-free conditions, confirming the formation of tight junctions. In cytokine-treated cultures, values dropped to approximately 180–220 Ω·cm^2^, consistent with increased permeability. These findings align with the concept of a “leaky gut” under inflammatory conditions and support the model’s relevance for simulating IBD-related epithelial barrier disruption. Its standardized, scalable format supports high-throughput testing of therapeutic candidates or CRISPR-based screens for immune regulators.

Given these characteristics, the cytokine-complemented model harboring M/DC precursors without RA pre-treatment may mirror the pathological immune activation seen in IBD, characterized by pro-inflammatory monocytes (CD14^+^CD16^+^) and elevated T cell activation markers such as CD25 and CD69 (summarized in Table 1). Introducing RA-primed M/DC precursors in this cytokine-complemented model slightly dampened T cell activation, potentially resembling moderate inflammation or “physiological inflammation” in the steady-state. In contrast, the cytokine-deficient model supported the presence of CD103^+^ DCs and CX3CR1^+^ macrophages, accompanied by CD45RO^+^ memory T cells without signs of activation. When established with M/DC precursors lacking RA priming, the cytokine-deficient model showed minimal T cell activation, likely due to the absence of regulatory DCs. This condition reflects a quiescent or suppressive state characterized by non-activated CD45RA^+^ T cells.

## 5. Conclusions

In conclusion, an advanced 3D small intestinal in vitro model was utilized to investigate immune cell differentiation and function. The results show that the epithelial cells within this model impose a suppressive influence on M/DC precursor cell differentiation. It was demonstrated that pro-inflammatory cytokines overcome this suppressive environment. RA priming of M/DC precursors significantly enhanced the differentiation of CD103^+^ DCs and CX3CR1^+^ macrophages. Such RA-primed cells were reduced in response to pro-inflammatory cytokines. This suggests that the chronic inflammatory signals in IBD may reduce the regulatory potential of RA, presenting a challenge in restoring immune balance within an inflamed environment. Taken together, the presented findings establish a controllable, human-specific platform to dissect cellular signaling cues and immune cell crosstalk in the context of intestinal inflammation. The novelty of this model lies in its capacity to integrate epithelial and immune components in a defined and modular system, enabling both inflammatory and tolerogenic responses to be studied within the same framework. We believe the human 3D intestinal model presented here will provide a valuable platform for understanding the dynamics of gut immune responses and for testing therapeutic interventions aimed at restoring mucosal integrity and immune tolerance in inflammatory diseases such as IBD.

## 6. Limitations of the Study

One limitation of the study is the absence of gene expression profiling for epithelial cells and immune cell populations. While our model suggests that these cells resemble key components of the human small intestine, including epithelial subtypes and immune subsets, this remains unverified at the transcriptomic level. To address this, parallel single-cell RNA sequencing will be performed to compare in vitro and in vivo cell types and states. This will allow us to assess the extent to which this setup replicates native intestinal cell populations by examining the expression of lineage-defining genes. In addition, transcriptomic data may enable the identification of novel markers for M cells, which remain poorly characterized in human systems.

Furthermore, functional validation of CD103^+^ DCs and CX3CR1^+^ macrophages—such as through Treg induction assays or phagocytic profiling—will be necessary to confirm their immunoregulatory roles.

Lastly, the presented model focuses on epithelial–immune signaling and does not include mesenchymal or stromal cells, which are known to provide additional regulatory cues in vivo. Future expansions may integrate stromal elements to further enhance model complexity and physiological relevance.

## Figures and Tables

**Table 1:** Similarities and differences between the four models, drawing parallels to inflammatory bowel disease (IBD) conditions.

*Modell*	*Immune Phenotype*	*T Cell Phenotype*	*Immune State*	*Main Finding*	*Inflammatory Context*
**Cytokine-complemented with RA-primed M/DC precursors**	CD14^++^, CD16^++^, CD103^+^, CX3CR1^-^	**Memory T cells (CD45RO^+^)** with signs of **activation (CD25^+^, CD69^+^)**	Moderate inflammation or “physiological inflammation”	Increased pro-inflammatory monocytes (CD14^+^CD16^+^), partially regulatory DCs (CD103^+^), but does not fully suppress. T cells are inflammation, activated (CD25^+^, CD69^+^).	RA modulates inflammation but does not fully counterbalance pro-inflammatory signals.
**Cytokine-complemented with untreated M/DC precursors**	CD14^++^, CD16^++^, CD103^-^	**Strong T cell activation** with high expression of **CD45RO^+^ (memory)** and **CD25^+^, CD69^+^ (active T cells)**	High, IBD-like inflammation	High pro-inflammatory monocyte activity (CD14^+^, CD16^+^), no CD103^+^ regulatory cells, strong T cell activation (CD25^+^, CD69^+^), leading to uncontrolled inflammation.	Uncontrolled inflammation, no regulatory modulation; model represents active, uncontrolled inflammation as seen in IBD, with a severe lack of immune regulation (CD103^+^ DCs are absent)
**Cytokine-deficient with RA-primed M/DC precursors**	CD103^++^, CX3CR1^++^	**Memory T cells (CD45RO^+^)**, with **no signs of activation (CD25^-^ CD69^-^)**	Low inflammatory state	RA-priming fosters immune tolerance, with abundant regulatory cells (CD103^+^, CX3CR1^+^). T cells show memory phenotype (CD45RO^+^) without activation.	RA promotes immune homeostasis, inducing regulatory cells and maintaining a balanced immune environment.
**Cytokine-deficient with untreated M/DC precursors**	Few monocytes/macrophages, CD103^-^	**Naive T cells (CD45RO^+^)** with **minimal activation (CD25^-^ CD69^-^)**	Immune quiescent or “suppressive state”	Epithelial cells maintain quiescence, minimal immune activation, few monocytes, no CD103^+^ cells, only naive T cells	Epithelial cells create an inhibitory milieu, preventing immune activation and maintaining quiescence.

## Data Availability

All data are included in the article and its Supplementary Materials. Further information is available from the corresponding author upon request.

## Supplementary Materials

The following supporting information can be downloaded at: https://www.mdpi.com/article/10.3390/cells14171300/s1, Table S1: Flow cytometry antibodies.
