# Supplementary material for: Retinoic Acid Modulates Immune Differentiation in a Human Small Intestinal In Vitro Model
Source: Cells. 2025 Aug 22;14(17):1300. doi: 10.3390/cells14171300 (PMC12428158; doi:10.3390/cells14171300)
Supplement: Supplementary file 1 [file cells-14-01300-s001.zip › cells-3799417-supplementary.pdf]

Table S1. Flow cytometry antibodies.

Antibody (anti-)	***Fluorochrome***	***Clone***	***Company***
CD3	APC-R700	SK7 (Leu-4)	BD Bioscience
CD4	APC	EDU-2	ImmunoTools
CD11b	Pe-Cy7	ICRF44	BioLegend
CD14	AF700	M5E2	BD Biosciences
CD16	BUV395	2A9-1	BD Biosciences
CD25	BV421	2A3	BD Bioscience
CD45RA	FITC	MEM-56	ImmunoTools
CD45RO	BV605	UCHL1	BD Bioscience
CD69	BV395	FN50	BD Bioscience
CD80	AF700	L307.4	BD Biosciences
CD83	APC	HB15e	BD Biosciences
CD86	Pe-Cy7	2331 (FUN-1)	BD Biosciences
CD103	BV421	LF61	BD Biosciences
CD103	Pacific Blue	LF61	BioRad
CD209	eAPC	eB-h209	Thermo Fisher Scientific
CX3CR1	BV750	2A9-1	BD Biosciences
HLA-DR	FITC	G46-6	BD Biosciences
Sirpα	FITC	15-414	BioLegend
